# *Plasmodium falciparum pfhrp2* and *pfhrp3* gene deletions among patients in the DRC enrolled from 2017 to 2018

**DOI:** 10.1038/s41598-021-02452-3

**Published:** 2021-11-26

**Authors:** Jessica N. McCaffery, Douglas Nace, Camelia Herman, Balwan Singh, Eric Mukomena Sompwe, Papy Mandoko Nkoli, Dieudonné Mumba Ngoyi, Gauthier Mesia Kahunu, Eric S. Halsey, Eric Rogier

**Affiliations:** 1 Malaria Branch, Division of Parasitic Diseases and Malaria, Centers for Disease Control and Prevention, Atlanta, GA 30329 USA; 2 School of Public Health, University of Lubumbashi, Lubumbashi, Democratic Republic of the Congo; 3 National Institute of Biomedical Research, Kinshasa, Democratic Republic of the Congo; 4 Department of Pharmacology and Therapeutics, Faculty of Pharmaceutical Sciences, University of Kinshasa, Kinshasa, Democratic Republic of the Congo; 5 US President’s Malaria Initiative, Atlanta, GA USA

**Keywords:** Epidemiology, Genotype

## Abstract

Rapid diagnostic tests (RDTs) detecting histidine-rich protein 2 (HRP2) and HRP3 are widely used throughout sub-Saharan Africa (SSA) to diagnose *Plasmodium falciparum* malaria. However, multiple SSA countries have reported *pfhrp2* and *pfhrp3* (*pfhrp2/3*) gene deletions. Blood samples (n = 1109) collected from patients with *P. falciparum* infection from six health facilities throughout the Democratic Republic of the Congo (DRC) from March 2017 to January 2018 were evaluated for *pfhrp*2/3 deletions. Samples were assayed for HRP2, pan-*Plasmodium* LDH (pLDH) and aldolase (pAldolase) antigens by bead-based multiplex antigen assay. Samples with low HRP2 concentration compared to pLDH and pAldolase antigens were selected for further *pfhrp2/3* genotyping PCRs. The majority of blood samples (93.3%, 1035/1109) had high concentrations of the HRP2 antigen. Single deletions of *pfhrp2* were identified in 0.27% (3/1109) of screened samples, with one sample from each of the Kapolowe, Mikalayi, and Rutshuru study sites. A *pfhrp3* single deletion (0.09%, 1/1109) was found in the Kapolowe site. Dual *pfhrp2* and *pfhrp3* deletions were not observed. Due to, the low numbers of *pfhrp2* deletions and the sporadic locations of these deletions, the use of HRP2-based RDTs appears to still be appropriate for these locations in DRC.

## Introduction

According to the World Health Organization, there were an estimated 215 million cases of malaria in Africa in 2019, accounting for just over 94% of the 228 million cases reported globally that year^1^. Of the 87 countries worldwide endemic for malaria in 2019, the Democratic Republic of the Congo (DRC) had the second-highest disease burden, with 12% of global cases and 11% of global deaths^1^, all of which were attributed to *Plasmodium falciparum*^2^. Assessment of the factors affecting malaria prevention, diagnosis, and treatment are currently underway to get DRC on track to meet the milestones laid out by the WHO’s Global Technical Strategy for malaria for 2025^1,3^.

In 2010, the WHO recommended that all patients with suspected malaria undergo parasitological confirmation by microscopy or rapid diagnostic test (RDT) before beginning treatment^4,5^. The result of these guidelines has been the widespread deployment of RDTs as they can be performed with limited training and return a test result within 15–20 min^5^. Of the 345 million RDTs sold annually, those based on *P. falciparum* histidine-rich protein 2 (HRP2) are the most common due to the high expression levels of this antigen and HRP2-based detection being more sensitive compared to pan-*Plasmodium* LDH (pLDH) detection^1,6^. HRP2-based RDTs diagnose *P. falciparum* infections via antibodies that bind to common epitopes present on the HRP2 and closely-related HRP3 antigens^7^.

Despite the advantages of HRP2-based RDTs, one of the most urgent threats to malaria diagnosis is the emergence of deletions in the *P. falciparum* histidine-rich protein 2 and 3 (*pfhrp2/3*) genes, which allows parasites to evade detection by these tests, contributing to false-negative results that can prevent appropriate case management of malaria patients. Deletions in the *pfhrp2/3* genes from parasites in nature were first reported in Peru in 2010^8^. There have since been reports of deletions in various countries, including multiple countries in Africa^9–14^. In 2019, the WHO recommended changing policy away from HRP2-based tests when the local prevalence of *pfhrp2* deletions in the parasite population reached ≥ 5%. This threshold is the breakpoint where the proportion of cases missed by the less sensitive non-HRP2-based tests would be lower than those missed by continued use of HRP2-based RDTs^15^. Past reports have found a low prevalence of *pfhrp2/3*-deleted isolates in DRC, though some sites were approaching (or exceeding) this 5% threshold^16,17^.

As part of the President’s Malaria Initiative-Supported Antimalarial Resistance Monitoring in Africa (PARMA) Network, therapeutic efficacy studies (TESs) are conducted periodically in partner countries, including DRC, to monitor the efficacy of antimalarial drugs and to determine the prevalence of antimalarial-resistant *P. falciparum* parasite populations^18^. As part of these studies, patients are enrolled at health facilities throughout the country following diagnosis of *P. falciparum* by microscopy and dried blood spot (DBS) samples are collected from participants on the day of enrollment and during follow-up^18^. Although these routine TESs are designed for estimation of drug efficacy and the prevalence of antimalarial-resistant parasite populations, they provide an opportunity to analyze local clinical *P. falciparum* infections for potential *pfhrp2/3* deletions. This current study reports the prevalence of *pfhrp2/3* deletions from samples collected in DRC from symptomatic, microscopy-confirmed *P. falciparum*-infected children under the age of five enrolled in a TES between March 2017 and January 2018.

## Methods

### Ethics statements and study sites

**Figure 1:**
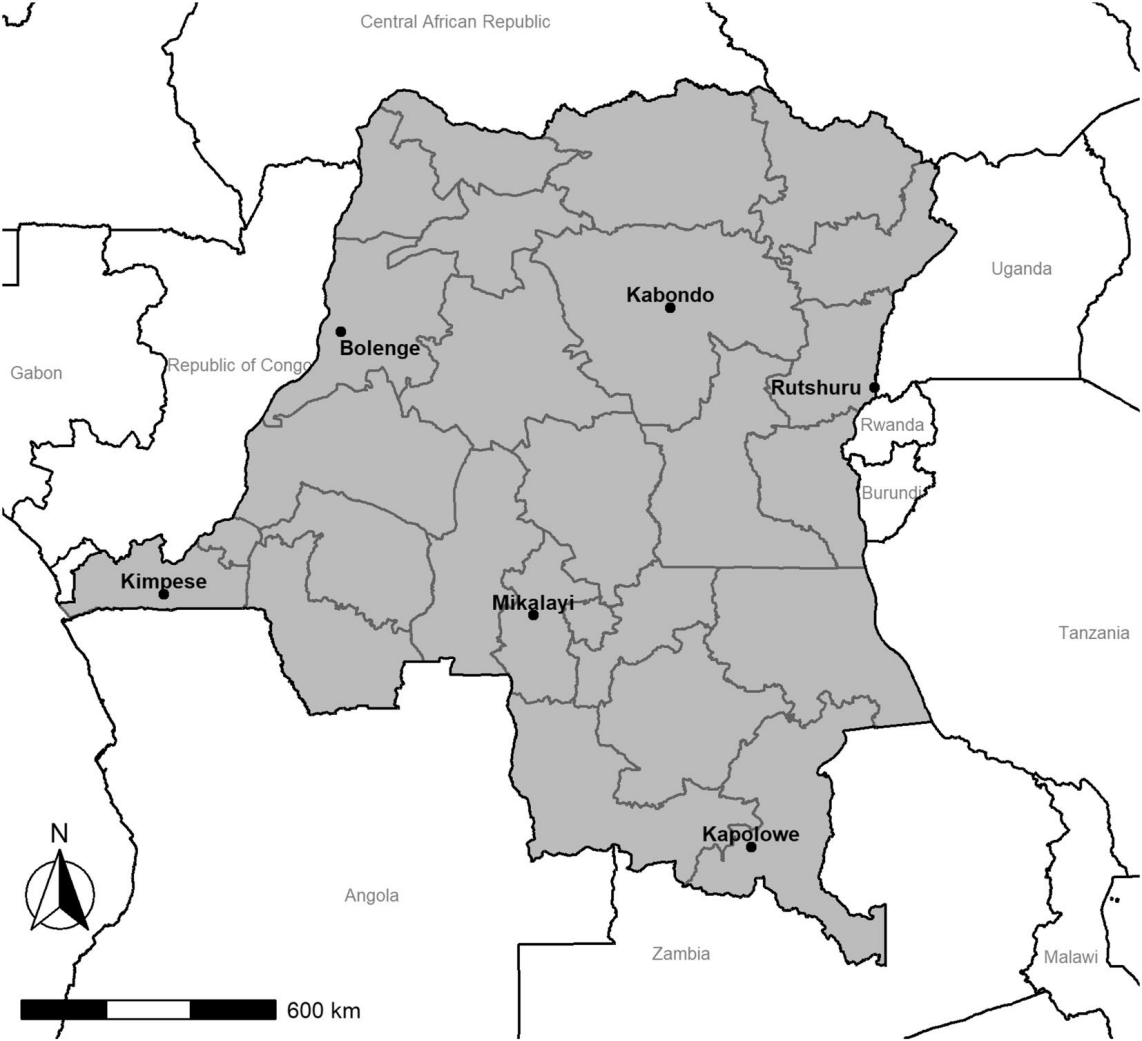
Location of antimalarial therapeutic efficacy monitoring sites, the Democratic Republic of the Congo (DRC), March 2017–January 2018. Samples were collected from six sites in DRC: Kabondo, a district of the city of Kisangini, in the northern province of Tshopo; Bolenge, located on the Congo River in the province of Equateur that borders the Republic of Congo; Rutshuru, in the mountainous province of North Kivu bordering Uganda and Rwanda; Kimpese, in the Kongo Central province that borders the Republic of Congo and Angola; Mikalayi, in the province of Kasai Central; and Kapolowe, situated on the shore of Lake Tshangalele in the tropical southern province of Haut Katanga bordering Zambia.

DBS used for this study were collected as part of a TES based on the standard WHO protocol^19^ designed to assess the efficacy of artemether-lumefantrine, artesunate-amodiaquine, and dihydroartemisinin-piperaquine in six sites representing different epidemiologic zones within DRC: Kabondo, a district of the city of Kisangani, in the northern province of Tshopo; Bolenge, located on the Congo River in the province of Equateur that borders the Republic of Congo; Rutshuru, in the mountainous province of North Kivu bordering Uganda and Rwanda; Kimpese, in the Kongo Central province that borders Angola; Mikalayi, in the province of Kasai Central; and Kapolowe, situated on the shore of Lake Tshangalele in the tropical southern province of Haut Katanga bordering Zambia (Fig. 1, prepared using R Version 4.0.1, R Foundation for Statistical Computing, Vienna, Austria, https://www.r-project.org/). Based on the 2017–2018 Multiple Indicator Cluster Survey published by the National Institute of Statistics (INS) of DRC^20^, the malaria RDT prevalence among children aged 6–59 months was reported to be 38.5% nationally and 52.2% in Tshopo, 45.5% in Kasai Central, 40.0% in Kongo Central, 11.4% in North Kivu, 43.7% in Haut Katanga, and 39.0% in Equateur Province. Based on the national and regional malaria RDT prevalence rates, these study sites should be representative of the nation as a whole for malaria transmission.

Study recruitment took place from March 2017 to January 2018. Ethical clearance for this study was provided by the DRC Ethics Committee of the School of Public Health of the University of Kinshasa, and the protocol was approved as a non-research program evaluation by the Office of the Associate Director for Science, Center for Global Health at Centers for Disease Control and Prevention (CDC; 2018-035). Patient recruitment was performed in accordance with the Declaration of Helsinki. Patients were only enrolled in this study if their parent or guardian chose to give consent. Informed consent forms were made available in French and translated into Lingala, Kikongo, Swahili, and Tshiluba, and read aloud in its entirety to parents and guardians to ensure understanding of the potential risks and benefits of the study. Parents or guardians who were unable to read or write were also given the opportunity to select study-independent co-signers for informed consent forms. All patient information was kept confidential, and an individual identification number was assigned to each sample for data entry. Staff from the CDC provided technical assistance^18^; this activity was reviewed by CDC and was conducted consistent with applicable federal law and CDC policy.

### Patient enrollment and sample collection

Children aged 6–59 months were recruited at participating health facilities following microscopy confirmed *P. falciparum* infection with parasite density between 2,000 and 200,000 trophozoites/µl and temperature of ≥ 37.5 °C. Patients were automatically excluded from the study if they showed the presence of any of the following conditions: mixed infection with other *Plasmodium* species, other severe illness, malnutrition, hemoglobin level less than 5 g/dL, hematocrit level less than 15%, or weight of less than 5 kg. Informed consent was obtained from the accompanying parent or guardian. Informed consent forms were available in French and translated into the following local languages: Kikongo, Lingala, Swahili, and Tshiluba. Upon enrollment (day 0), medical history was recorded, and capillary blood samples were collected on 903 Whatman filter paper (GE Healthcare, Chicago, IL). Of the 1613 enrolled participants, 1109 DBS samples from day 0 were provided to US CDC in Atlanta for antigen detection assays and subsequent *pfhrp2/3* genotyping if applicable.

### Bead-based antigen multiplex assay

A bead-based multiplex antigen detection assay was used to determine the levels of pan-*Plasmodium* pAldolase and pLDH, and *P. falciparum* HRP2 antigens as previously described^21–23^. A complete description of this assay as performed for this study is included in the Supplemental Methods. Antigen profiles generated by multiplex assay were used to establish samples with atypically low HRP2 levels compared to either pan-*Plasmodium* antigen. Because the HRP2 signal is obtained from the binding of antibodies to multiple repeat epitopes present in both HRP2 and HRP3 proteins, both antigens contribute to the HRP2 assay signal. Additionally, for wild-type *P. falciparum* isolates, the assay signal for HRP2 is higher than either pan-*Plasmodium* antigen since the HRP2 protein is more highly expressed throughout the erythrocytic stage of infection and HRP2 persists in the blood for several weeks following antimalarial treatment^7^. Using a strategy described previously^23,24^ samples were classified as HRP2 low (or HRP2 negative) based on the relationship between the two pan-*Plasmodium* antigens (aldolase and LDH) and the HRP2/3 signal and were selected for further characterization by genetic assays because these samples show phenotypic evidence of potential *pfhrp2* and/or *pfhrp3* deletions.

### DNA extraction

For selected samples, genomic DNA was extracted from DBS by column-based purification. A single 6 mm hole punch was taken from each selected DBS and processed using the QIAamp Blood Mini Kit (Qiagen Inc.) according to the manufacturer’s protocol for DNA purification from DBS. Extracted DNA was stored at 4 °C for immediate use or at − 20 °C for long-term (> 4 weeks) storage.

### Confirmation of *P. falciparum* infection by photo-induced electron transfer polymerase chain reaction

Photo-induced electron transfer polymerase chain reaction using fluorogenic primers (PET-PCR), was performed to confirm the presence of *Plasmodium* genus and *P. falciparum* DNA using the extracted genomic DNA^25^. A full description of primers, master mix preparation, and PCR conditions used for this study are included in the Supplemental Methods.

### PCR assays for *pfmsp1*, *pfmsp2*, *pfhrp2*, and *pfhrp3* genotyping

Following the confirmation of the presence of *Plasmodium* genus and *P. falciparum* DNA by PET-PCR, samples were further screened for DNA quality by nested PCR for *pfmsp1* and *pfmsp2* single-copy genes^26^. Samples that failed to amplify by *pfmsp1* or *pfmsp2* were omitted from further genotyping analysis because DNA quality (or quantity) was assumed to be compromised. A one-step PCR was used to determine the presence of the *pfhrp2* gene^27^, and two separate nested PCRs were used for the confirmation of the presence of the *pfhrp3* gene: a *pfhrp3* exon 1–2 PCR and an exon 2 PCR^22,26^. Deletion of the *pfhrp3* gene was confirmed if the sample was unable to amplify the exon 1–2 PCR product. For all genotyping PCR assays, any samples that produced discordant results after two runs were subjected to a third run to obtain final tie-breaker results. The full description of these genotyping assays as performed here is included in the Supplemental Methods. For all PCRs, *P. falciparum* DNA controls 3D7, 7G8, Hb3, Dd2, and a water non-template control were used. The presence or absence of the sequence of interest in the final PCR product was confirmed by gel electrophoresis.

## Results

### Demographics of participants

DBS samples obtained from 1109 participants on the day of enrollment were available for evaluation of antigen profile and *pfhrp2*/*3* deletions, with participant information outlined in Table 1. Participant enrollment was fairly evenly distributed among the six study sites, with the lowest number of children enrolled in Rutshuru with 134 (12.1%) participants; the study sites in Kabondo and Kapolowe both enrolled the highest numbers of participants with 218 (19.7%) each. Kimpese, Bolenge, and Mikalayi provided the remaining 194 (17.5%), 174 (15.7%), and 171 (15.4%) of participants, respectively.

**Table 1:** Demographics of study participants and selection of specimens with atypical HRP2 antigen levels for further analysis.

Category	n
Total participants	1109
**Study site (City/Town, Province)**	
Bolenge, Equateur	174 (15.7%)
Kabondo District, Kisangini, Tshopo	218 (19.7%)
Kapolowe, Haut Katanga	218 (19.7%)
Kimpese, Kongo Central	194 (17.5%)
Mikalayi, Kasai Central	171 (15.4%)
Rutshuru, North Kivu	134 (12.1%)
**Sex**	
Male	546 (49.2%)
Female	535 (48.2%)
Not listed	28 (2.5%)
**Age (months)**	
Age Range	6 to 59
Age Mean	31.4

Gender information was included for all but 28 (2.5%) of the participants. The gender ratio of the participants with recorded gender data was even, with 49.2% of the participants being male and 48.2% of the participants being female. The median age of participants was 31 months, with a mean of 31.4 months.

### Multiplex antigen detection

**Figure 2:**
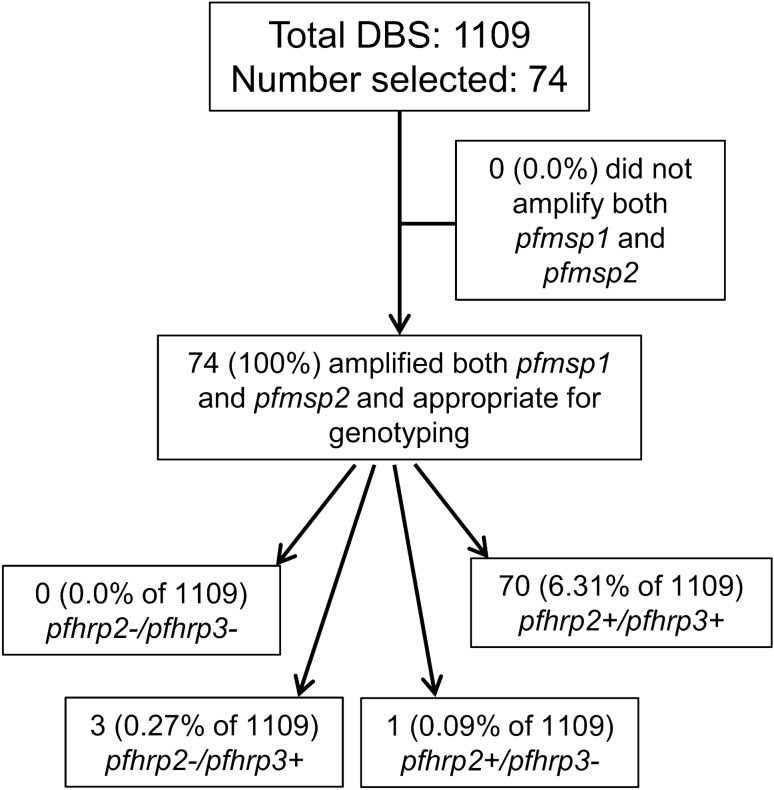
Sample processing and results for *pfhrp2* and *pfhrp3* genotyping in the Democratic Republic of the Congo. Results presented as a flowchart for the entire sample set available for antigen screening. Terminal boxes show the presence ( +) or absence ( −) of the *pfhrp2* and *pfhrp3* genes from samples appropriate for genotyping. All percentages shown are out of the total 1109 DBS samples analyzed.

All 1109 DBS were first subjected to multiplex antigen detection to assess the antigen levels of HRP2 (also detecting HRP3), pAldoase, and pLDH. Using low HRP2 assay signal as our selection criteria as described in Methods, we selected a total of 74 (6.7% of all) samples for assessment of *pfhrp2/3* genotype. Figure 2 shows a flow chart detailing sample processing from initial selection to the final *pfhrp2/3* genotyping results.

**Figure 3:**
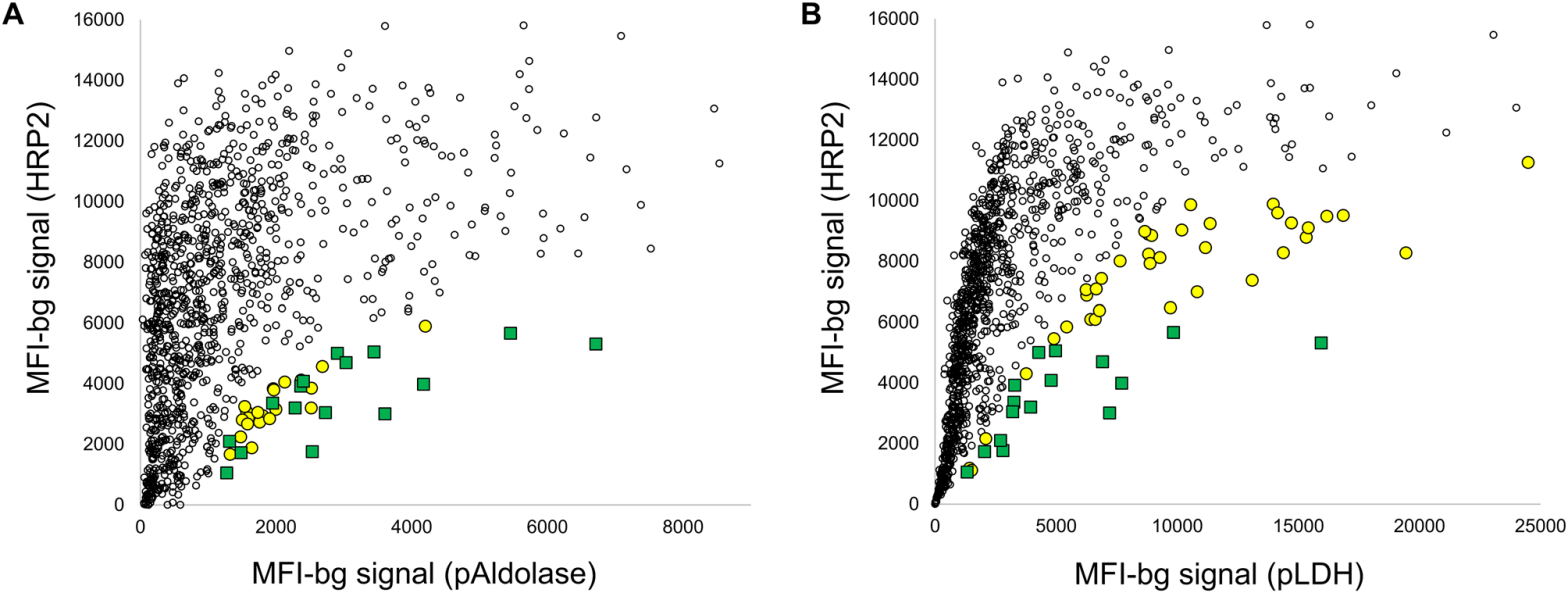
Multiplex antigen detection and sample selection for genetic assays. Scatter plots comparing the MFI-bg signal for pAldolase (panel **A**) or pLDH (panel **B**) antigen signal on the x-axis in comparison with HRP2 assay signal on the y-axis. Yellow circles indicate samples selected for either pAldolase or pLDH, green circles indicate samples selected based on both pan-*Plasmodium* markers.

The number of specimens selected from each study site is summarized in Table 2. Of the 74 samples selected across all six study sites, 21 were selected based on low HRP2 signal in comparison to pAldolase signal, 37 were selected based on low HRP2 signal in comparison with pLDH signal, and 16 were selected because they showed low HRP2 signal compared to the signal generated for both antigens. Scatterplots displaying the antigen signal for HRP2 in comparison with pAldolase and pLDH are shown in Fig. 3, with samples selected for further analysis based on a single antigen or both antigens indicated. The study site from which the highest number of selected samples was Mikalayi with 26 samples, and Kabondo had the second most samples selected at 18. The study site with the lowest number of selected samples was Kimpese, with five.

**Table 2:** Selection of samples for further genetic analysis by study site.

Study site	Number specimens at enrollment	Number specimens selected for genetic assays (%)	Number selected on pAldolase:HRP2 ratio only	Number selected on pLDH:HRP2 Ratio Only	Number selected on ratio to both antigens
**Total**	1109	74 (6.7%)	21 (1.9%)	37 (3.3%)	16 (1.4%)
Bolenge	174	7 (4.0%)	1	3	3
Kabondo	218	18 (8.3%)	6	10	2
Kapolowe	218	9 (4.1%)	7	1	1
Kimpese	194	5 (2.6%)	2	1	2
Mikalayi	171	26 (15.2%)	3	19	4
Rutshuru	134	9 (6.7%)	2	3	4

### Genotyping for *pfmsp1, pfmsp2, pfhrp2,* and *pfhrp3*

Testing of genomic DNA by PET-PCR confirmed the presence of *P. falciparum* DNA in all 74 of the selected samples. All 74 selected samples successfully amplified both *pfmsp1* and *pfmsp2* and were therefore appropriate for reporting of *pfhrp2/3* genotype (Fig. 2).

PCR reactions determined that 70 of the 74 selected samples showed a wild-type genotype of *pfhrp2* + */pfhrp3* + . A *pfhrp2-/pfhrp3* + single deletion genotype was found in three samples, or 0.27% of the 1109 total samples. We observed a single sample with a genotype of *pfhrp2* + */pfhrp3-* (0.09%). No *pfhrp2-/pfhrp3-*dual deletions were found among all samples genotyped.

## Discussion

HRP2-based RDTs play a critical role in the diagnosis of *P. falciparum* malaria throughout sub-Saharan Africa. Due to the many advantages of HRP2-based RDTs, a significant prevalence of *pfhrp2/3* deletions in an area could have a substantial impact on *P. falciparum* case management, control, and elimination. DBS samples collected as part of routine TES provide convenience sampling of microscopy-confirmed *P. falciparum* infections in which to investigate HRP2 expression levels in endemic parasites and potential deletions in the *pfhrp2/3* genes. However, for more precise deletion prevalence estimates, WHO guidance^28^ recommends enrolling participants from at least ten health facilities per province. Since this current study included samples from an antimalarial TES that did not enroll the WHO recommended number of health centers for *pfhrp2* deletion estimates, the data presented here is not meant to serve as a precise prevalence estimate for *pfhrp2/3* gene deletions in DRC but instead provide an additional signal for the presence and patterns of these deletions throughout the country. As TES are conducted every two to 3 years within DRC, future TESs will expand on the findings presented here to provide longitudinal data on deletion genotypes that are present across the country.

In total, 1109 DBS collected from participants on the day of enrollment from the six sites across DRC were made available for quantitative antigen detection and genotypic analysis. The use of multiplex antigen detection assays to quantify the levels of HRP2 antigens compared to two essential pan-*Plasmodium* antigens^23^ allowed for the selection of samples by phenotypic criteria with abnormally low HRP2 antigen levels, thereby saving time and resources that would have been required to perform genotypic assays for all 1109 samples. The use multiplex antigen detection provides a high-throughput, sensitive antigen screen that allows for identification of samples with low HRP2/3 expression requiring *pfhrp2/3* deletion genotyping^22,23,29–31^ and assays for the actual antigen target of the HRP2-based RDTs. Other laboratory assays are also available which may be able to provide sensitive multiplex data to screen for potential *pfhrp2/3* deletions by antigen profiles^32,33^.

Assessment of the *pfhrp2/3* genotype of the 74 samples selected based on low or absent HRP2 levels revealed three samples with a genotype of *pfhrp2-/pfhrp3* + and one sample with a genotype of *pfhrp2* + */pfhrp3-*, with no dual *pfhrp2/3* deletions observed. This data shows that 0.27% of isolates from *P. falciparum* infected children enrolled in this TES were deleted for *pfhrp2,* and 0.09% were deleted for *pfhrp3.* Importantly, we observed the three *pfhrp2* deletions from three separate study sites, Kapolowe, Mikalayi, and Ruthsuru, and one *pfhrp3* deletion from Kapolowe, suggesting that these deletions are both at low prevalence in clinical infections, but that these deletions are also located sporadically throughout the country without evidence for clustering.

By utilizing the antigen screen methodology to phenotypically select samples for genotyping, it is possible this strategy may underestimate the true number of *pfhrp2* or *pfhrp3* deletions from these clinical infections. It is also possible individuals could have been infected with multiple *P. falciparum* haplotypes, resulting in the masking of deleted genotypes by high levels of HRP2 in the blood sample (which would also occur if screened by an HRP2-based RDT). Additionally, residual HRP2 antigen from a previous wild-type *P. falciparum* infection could have masked the phenotype of a *pfhrp2/3* deleted parasite from the current infection. However, one advantage of this sensitive antigen detection strategy is the ability to receive a quantitative result for the HRP2 signal rather than a simple binary result through an RDT. Even if a sample is “HRP2 positive,” if the expression levels are notably lower given the levels of pAldolase and pLDH, the sample could be flagged as suspect and genotyped accordingly^22,23^.

The *pfhrp2* deletions in three out of 1109 (0.27%) microscopy-confirmed *P. falciparum*-infected symptomatic children under five reported in this current study are much lower than estimates of *pfhrp2* deletions from a previous cross-sectional DRC survey of non-treatment seeking children under five enrolled at households from 2013 to 2014. That study estimated a nationwide prevalence of 5.4% for *pfhrp2-*deleted *P. falciparum* parasites with no observed *pfhrp3* deletions^17^. However, the findings of sparse numbers of deletions presented in this current study are consistent with a more recent DRC study which enrolled 3627 subjects with symptomatic malaria in 2017 and found eight isolates with suspected *pfhrp2/3* deletions that were later excluded after whole-genome sequencing revealed intact *pfhrp2* and *pfhrp3* genes^16^. The authors concluded that these findings support the continued use of HRP2-based RDTs in the region, but emphasized that careful laboratory workflows are necessary for *pfhrp2/3* gene deletion analyses.

Overall, DBS collected from TESs provide a useful convenience sampling method with specimens collected routinely from symptomatic patients with microscopy-confirmed *P. falciparum* infection. While not able to provide precise prevalence estimates for an entire country, the phenotypic and genotypic data generated from TES samples may provide evidence of deleted isolates within a particular area and allow for more efficient follow-up using surveys specifically aimed at determining the accurate prevalence of *pfhrp2/3* deletions^28^. This report provides strong evidence that *P. falciparum* infections from these six sites in DRC show high levels of HRP2 and HRP3 expression. Assessment of phenotypic and genotypic data from future TES samples will continue to provide insights into the presence of *pfhrp2/3* deletions in DRC and, if a higher prevalence is noted later, may justify more targeted surveys to establish precise prevalence estimates.

## Supplementary Information

Supplementary Information.

## Data Availability

The datasets used and/or analyzed during the current study are available from the corresponding author on reasonable request.

## References

[1] WHO. (World Health Organization, Geneva, 2019).

[2] WHO. 3 (World Health Organizatoin, Geneva, Switzerland, 2018).

[3] WHO. *Global technical strategy for malaria 2016–2030*. 29 p. (World Health Organization, 2015).

[4] WHO. (World Health Organization, Geneva, 2010).

[5] WHO. *Universal Access to Malaria Diagnostic Testing: An Operational Manual*. 4–6 (World Health Organization, 2011).

[6] Li B (2017). Performance of pfHRP2 versus pLDH antigen rapid diagnostic tests for the detection of *Plasmodium falciparum*: a systematic review and meta-analysis. Arch. Med. Sci..

[7] Poti KE, Sullivan DJ, Dondorp AM, Woodrow CJ (2020). HRP2: Transforming malaria diagnosis, but with caveats. Trends Parasitol..

[8] Gamboa D (2010). A large proportion of *P. falciparum* isolates in the Amazon region of Peru lack pfhrp2 and pfhrp3: implications for malaria rapid diagnostic tests. PLoS ONE.

[9] Beshir KB (2017). Plasmodium falciparum parasites with histidine-rich protein 2 (pfhrp2) and pfhrp3 gene deletions in two endemic regions of Kenya. Sci. Rep..

[10] Bharti PK (2016). Prevalence of pfhrp2 and/or pfhrp3 gene deletion in *Plasmodium falciparum* population in eight highly endemic states in India. PLoS ONE.

[11] Bosco AB (2020). Molecular surveillance reveals the presence of pfhrp2 and pfhrp3 gene deletions in *Plasmodium falciparum* parasite populations in Uganda, 2017–2019. Malar. J..

[12] Berzosa P (2020). First evidence of the deletion in the pfhrp2 and pfhrp3 genes in Plasmodium falciparum from Equatorial Guinea. Malar. J..

[13] Dorado EJ (2016). Genetic characterisation of plasmodium falciparum isolates with deletion of the pfhrp2 and/or pfhrp3 genes in Colombia: The Amazon Region, a challenge for malaria diagnosis and control. PLoS ONE.

[14] Berhane A (2018). Major threat to malaria control programs by plasmodium falciparum lacking histidine-rich protein 2, Eritrea. Emerg. Infect. Dis..

[15] WHO. (World Health Organization, Geneva, Switzerland, 2019).

[16] Parr JB (2021). Analysis of false-negative rapid diagnostic tests for symptomatic malaria in the Democratic Republic of the Congo. Sci. Rep..

[17] Parr JB (2017). Pfhrp2-deleted *Plasmodium falciparum* parasites in the Democratic Republic of the Congo: a national cross-sectional survey. J. Infect. Dis..

[18] Halsey ES (2017). Capacity development through the US President's malaria initiative-supported antimalarial resistance monitoring in Africa network. Emerg. Infect. Dis..

[19] WHO. 90 (World Health Organization, Geneva, Switzerland, 2009).

[20] Institut national de la statistique, I. (Kinshasa, République Démocratique du Congo, 2020)

[21] Rogier E (2017). Bead-based immunoassay allows sub-picogram detection of histidine-rich protein 2 from Plasmodium falciparum and estimates reliability of malaria rapid diagnostic tests. PLoS ONE.

[22] Herman C (2019). Multiplex malaria antigen detection by bead-based assay and molecular confirmation by PCR shows no evidence of Pfhrp2 and Pfhrp3 deletion in Haiti. Malar. J..

[23] Plucinski MM (2018). Screening for Pfhrp2/3-deleted *Plasmodium falciparum*, non-falciparum, and low-density malaria infections by a multiplex antigen assay. J. Infect. Dis..

[24] Bakari C (2020). Community-based surveys for *Plasmodium falciparum* pfhrp2 and pfhrp3 gene deletions in selected regions of mainland Tanzania. Malar. J..

[25] Lucchi NW (2013). Molecular diagnosis of malaria by photo-induced electron transfer fluorogenic primers: PET-PCR. PLoS ONE.

[26] Abdallah JF (2015). Prevalence of pfhrp2 and pfhrp3 gene deletions in Puerto Lempira, Honduras. Malar. J..

[27] Jones S (2020). One-step PCR: A novel protocol for determination of pfhrp2 deletion status in Plasmodium falciparum. PLoS ONE.

[28] WHO. (Geneva, Switzerland, 2020).

[29] van den Hoogen LL (2021). Rapid screening for non-falciparum malaria in elimination settings using multiplex antigen and antibody detection: post hoc identification of plasmodium malariae in an infant in Haiti. Am. J. Trop. Med. Hyg..

[30] Oviedo A (2020). Combination of serological, antigen detection, and DNA data for *Plasmodium falciparum* provides robust geospatial estimates for malaria transmission in Haiti. Sci. Rep..

[31] Lu A (2020). Screening for malaria antigen and anti-malarial IgG antibody in forcibly-displaced Myanmar nationals: Cox's Bazar district, Bangladesh, 2018. Malar. J..

[32] Martiáñez-Vendrell X (2020). Quantification of malaria antigens PfHRP2 and pLDH by quantitative suspension array technology in whole blood, dried blood spot and plasma. Malar. J..

[33] Jang IK (2020). Multiplex human malaria array: quantifying antigens for malaria rapid diagnostics. Am. J. Trop. Med. Hyg..

