# Supplementary material for: Plasmodium falciparum pfhrp2 and pfhrp3 gene deletions among patients in the DRC enrolled from 2017 to 2018
Source: Sci Rep. 2021 Nov 26;11:22979. doi: 10.1038/s41598-021-02452-3 (PMC8626453; doi:10.1038/s41598-021-02452-3)
Supplement: Supplementary file 1 — Supplementary Information. [file 41598_2021_2452_MOESM1_ESM.docx]

**Supplemental Methods**

**Deletions of the *Plasmodium falciparum pfhrp2* and *pfhrp3* genes among patients enrolled at six locations in the DRC: 2017–2018**

Jessica N. McCaffery^1^, Douglas Nace^1^, Camelia Herman^1^, Balwan Singh^1^, Eric Mukomena Sompwe^2^, Papy Mandoko Nkoli^3^, Dieudonné Mumba Ngoy^3^, Gauthier Mesia Kahunu^4^, Eric S. Halsey^1,5^, Eric Rogier^1^*.

**Author’s Information:**

^1^Malaria Branch, Division of Parasitic Diseases and Malaria, Centers for Disease Control and Prevention, Atlanta, GA, 30329, USA.

^2^ School of Public Health, University of Lubumbashi, DRC

^3^National Institute of Biomedical Research, Kinshasa, DRC

^4^ Department of Pharmacology & Therapeutics, Faculty of Pharmaceutical Sciences, University of Kinshasa, DRC

^5^ US President’s Malaria Initiative, Atlanta, GA, USA

**Antigen Multiplex Serology**

DBS samples were provided on Whatman 903 Protein Saver Card. 6 mm hole punches were taken from each sample and eluted to a final concentration of 1:20 in 200 µl of blocking buffer (Buffer B) composed of 0.5% polyvinyl alcohol (Millipore-Sigma, P8136), polyvinylpyrrolidone (Millipore-Sigma, PVP360), 0.5% Bovine Serum Albumin (Millipore-Sigma A4503), 0.3% Tween20 (Fisher BP337), 0.1% casein (Thermo Scientific, 37528), 0.05% sodium azide, and 3ug/mL of *E. coli* extract (included to prevent non-specific binding).

To assess the levels of individual antigens, three unique bead regions were individually coated with EDC/Sulfo-NHS intermediate reaction (1) using separate antibodies for each antigen targeted for capture: *Plasmodium* aldolase (12.5ug/12.5x10^6^ beads, rabbit IgG anti-aldolase, Abcam, Cambridge, UK; ab207494), *Plasmodium* LDH (12.5ug/12.5x10^6^ beads, mouse IgG anti-LDH, BBI Solutions, Cardiff, UK; BM355-Z8F7), and *P. falciparum* PfHRP2 (20ug/12.5x10^6^ beads, mouse IgG anti-HRP2, Abcam; ab9206).

Following antigen coupling to microbeads and elution of the DBS in Buffer B for at least 24 hours, a mixture containing 5 µl of each bead region was prepared in 5 mL of Buffer A, a PBS 1X buffer containing 0.5% BSA, 0.05% Tween20, and 0.02% sodium azide. A volume of 50 µl of the bead mixture was added to each well of the assay plate to obtain ~1500 beads per bead region per well. The assay plate was then placed on a Luminex magnet and incubated for one minute at room temperature (RT) before being gently tapped for 30 seconds. The liquid was evacuated into the sink, and the assay plate was washed three times with 100 µl of wash buffer consisting of PBS 1x with 0.05% Tween20, with the assay plate tapped 45 seconds between each evacuation and wash. Initial bead washing was followed by the addition of 50 µl of eluted sample to appropriate wells, with each sample run in duplicate. Assay plates were incubated for 90 mins at RT in the dark under gentle shaking.

Plates were washed three times, as before, and a mixture of detection antibodies was prepared in 5 mL of Buffer A using 2.5 µl of anti-pAldolase (1:1000x, rabbit anti-aldolase, Abcam; ab207494), 10 µl of anti-LDH (1:500x of 2:1:1 mixture [BBI Solutions BM355-P4A2:BioRad Pv-pLDH HCA156:BioRad Pf-pLDH HCA158]), and anti-HRP2 (1:500x, mouse IgG anti-HRP2, Abcam, ab9203). All three detection antibodies were biotinylated using the Thermo Scientific EZ-Link Micro Sulfo-NHS-Biotinylation Kit (ThermoFisher Scientific) according to the manufacturer’s protocol before beginning the multiplex assay. A volume of 50 µl of the detection antibody mixture was added to each well of the assay plate, and the plate was incubated at RT for 45 minutes with gentle shaking before being washed three times.

A volume of 50 µl of streptavidin-phycoerythrin (Invitrogen, Carlsbad, CA) diluted 1:200 in Buffer A was added to each well, and the assay plates were incubated for 30 minutes at RT with gentle shaking before washing three times. A volume of 50 µl of Buffer A was then added to each well and allowed to incubate for 30 minutes at RT with gentle shaking to reduce background signal from the streptavidin-phycoerythrin incubation. The assay plate was washed once more, and beads were resuspended in 100 µl of PBS and shaken for 30 seconds prior to being read on a Bio-Plex 200 instrument (BioRad, Hercules, CA). A target of 50 beads was read per bead region to obtain a median fluorescence intensity signal (MFI) for each analyte. Because each sample was run in duplicate, the mean of the MFIs among duplicates is presented. A final measure of MFI-background (MFI-bg) was obtained by subtracting MFI values for experimental wells by the MFI values from beads on each plate only exposed to the sample diluent (Buffer B) during the sample incubation step. In addition to two buffer control wells, each plate also included two wells each of 1:20 whole blood samples from a malaria naïve donor and a positive control consisting of a mixture of the recombinant antigens targeted by the antibodies bound to the beads. For the positive control antigens, recombinant pLDH and PfHRP2 antigens were provided by Microcoat Biotechnologie GmbH (Bernried, Germany) and lyophilized preparations were rehydrated according to the manufacturer’s instructions. The *Plasmodium vivax*-specific isoform of aldolase was produced at the CDC, as described previously (2).

**Confirmation of *P. falciparum* infection by photoelectron-induced electron transfer PCR**

Photo-induced electron transfer polymerase chain reaction using fluorogenic primers, known as PET-PCR, was performed to confirm the presence of *Plasmodium* Genus and *P. falciparum* DNA using the extracted genomic DNA, performed as described by Lucchi et al. (3). Briefly, the PET-PCR reaction master mix was prepared per reaction using 3.25 µl H_2_O, 10 µl pf 2X TaqMan Environmental Master Mix Buffer (Thermo Fisher Scientific), and the following primer volumes from 10 µM preparations: 0.5 µl of the Genus forward primer (5’ GGC CTA ACA TGG CTA TGA CG 3’), 0.5 µl of the FAM-labeled Genus reverse primer (FAM- agg cgc ata gcg cct gg CTG CCT TCC TTA GAT GTG GTA GCT, where the target-independent tail is underlined), 0.5 µl of *P. falciparum* forward primer (5’ ACC CCT CGC CTG GTG TTT TT 3’), and 0.25 µl HEX-labeled *P. falciparum* reverse primer (HEX- agg cgc ata gcg cct gg TCG GGC CCC AAA AAT AGG AA). A volume of 15 µl was added to each reaction tube of an 8x optical tube strip (Agilent Technologies), and 5 µl of genomic DNA was added to appropriate reaction tubes, with all samples run in duplicate. DNA extracted from *P. falciparum* 3D7 culture, and a water non-template control were run in duplicate as controls. The PET-PCR reaction was run on a Stratagene Mx3005P (Agilent Technologies), and PCR cycle conditions were set to one cycle of 95°C for 15 minutes followed by 45 cycles of 95°C for 20 seconds, 63°C for 40 seconds, and 72°C for 30 seconds with the fluorescence signal read at the 63°C plateau. The fluorochrome ROX was set as the fluorescent control, and a cycle threshold (CT) value of 0.03 was used as a cutoff for HEX and FAM amplification curves. CT values above 40 were considered DNA negative, and CT values below 40 were considered DNA positive.

**PCR assays for *pfmsp1*, *pfmsp2*, *pfhrp2*, and *pfhrp3* genotyping**

Following the confirmation of the presence of *Plasmodium* genus and *P. falciparum* DNA by PET-PCR, samples were further screened for DNA quality by nested PCR for *pfmsp1* and *pfmsp2* single-copy genes (4). Samples that failed to amplify by *pfmsp1* or *pfmsp2* were omitted from further genotyping analysis because DNA quality was assumed to be compromised.

Briefly, the *pfmsp1* master mix was prepared per reaction using 7.5 µl of Promega 2X buffer (Promega, Madison, WI), 0.5 µl of the MSP1 outer forward (M1OF) primer (5’ CTA GAA GCT TTA GAA GAT GCA GTA TTG 3’), 0.5 µl of the MSP1 outer reverse (M1OR) primer (5’ ATT CTA ATT CAA GTG GAT CAG 3’), and 5.5 µl of H_2_O. Primer volumes are based on 10 µM preparations for all primers for the MSP1 and MSP2 nested PCRs. A volume of 14 µl was added to each reaction tube, and 1 µl of genomic DNA was added to the appropriate tubes. Each sample was run individually and performed by two independent operators for a total of two reactions. The primary PCR conditions for MSP1 were 95°C for five minutes, followed by 30 cycles of 95°C for 30 seconds, 51°C annealing step for 30 seconds, and 68°C for one minute. The reaction was then brought to 68°C for five minutes and then held at 4°C indefinitely until the samples could be retrieved. The MSP1 secondary reaction master mix was then prepared per reaction the same as for the primary reaction except the reverse primer was switched for the MSP1 inner reverse (M1IR) primer (5’ CAT ATC CAT CAA TTA AAT ATT TGA AAC C 3’), and the volume of water was reduced to 4.5 µl per reaction. A volume of 13 µl of the MSP1 secondary master mix was added to each of the reaction tubes, and 2 µl of the primary PCR product from the primary reaction was added to the corresponding secondary reaction tubes. The MSP1 secondary PCR conditions were the same as used for the MSP1 primary PCR except that the annealing temperature was increased to 52°C. The PCR master mix preparation and cycle conditions for *pfmsp2* primary and secondary reactions were the same as those used for *Pfmsp1* except that the MSP2 outer forward (M2OF) primer (5’ GAA GGT AAT TAA AAC ATT GTC 3’) and MSP2 outer reverse (M2OR) primer (5’ GAT GTT GCT GCT CCA CAG 3’) and an annealing temperature of 50°C was used for the primary reaction, and the MSP2 inner forward (M2IF) primer (5’ GAG TAT AAG GAG AAG TAT G 3’) and MSP2 inner reverse (M2IR) primer (5’ CTA GAA CCA TGA ATA TGT CC 3’) and annealing temperature of 48°C was used for the secondary reaction.

Following confirmation of the presence of *pfmsp1* and *pfmsp2*, a recently described one-step PCR was used to determine the presence of *pfhrp2* (5). Briefly, the one-step master mix was prepared per reaction using 10 µl of Q5 5X Buffer (New England Biolabs), 1 µl of 10 mM dNTPs (New England Biolabs), 0.63 µl of forward primer (Bravo_f1: ATG ATT CAT TAT TCT ATA TTT ATA AGG AAG ATT AC) and 0.63 µl of reverse primer (Bravo_r1: CACTTCATGTATTTATGTATGCAGAAC), 0.5 µl of Q5 high-fidelity enzyme (New England Biolabs), and 32.2 µl of water. Primer volumes were based on a concentration of 10 µM. A volume of 45µl of *pfhrp2* master mix was then added to each reaction tube, followed by 5 µl of genomic template DNA to the appropriate tubes. PCR cycle conditions for the *pfhrp2* one-step reaction were as follows: 98°C for three minutes, then 30 cycles of 98°C for 30 seconds, 60°C for one minute and 30 seconds, and 68°C for two minutes, followed by five minutes at 68°C, then the temperature was held at 4°C indefinitely until the samples could be retrieved.

Two separate nested PCRs were used for the confirmation of the presence of *pfhrp3*, an HRP3 exon 1-2 PCR, and an HRP3 exon 2 PCR, which have both been described previously (4, 6). For the *pfhrp3* exon 1-2 reaction, the primary master mix was prepared per reaction using 2 µl of Expand High Fidelity PCR Buffer (Millipore-Sigma), 2 µl of 2 mM dNTPs (New England Biolabs), 1 µl of HRP3 Exon 1-2 forward primary reaction (3E12F1) primer (5’ GGT TTC CTT CTC AAA AAA TAA AA 3’), 1 µl of HRP3 exon 1-2 reverse primary reaction (3E12R1) primer (5’ CCT GCA TGT GCT TGA CTT TA 3’), 0.2 µl of Expand High Fidelity Enzyme (Millipore Sigma), and 11.8 µl of water. Primer volumes were based on 15 µM preparations for both primary and secondary reactions for the *pfhrp3* exon 1-2 and exon 2 PCRs. A volume of 18 µl was aliquoted to each reaction tube, followed by 2 µl of genomic DNA template to the appropriate wells. The following PCR conditions were used for the *pfhrp3* exon 1-2 primary reaction: 95°C for five minutes, followed by 30 cycles of 95°C for 30 seconds, an annealing step of 53°C for 30 seconds, 68°C for 30 seconds, followed by five minutes at 68°C before the temperature was held at 4°C indefinitely until the samples could be retrieved. The primary reaction products were diluted 1:10 in water before proceeding to the secondary reaction. The *pfhrp3* exon 1-2 secondary reaction master mix was prepared the same as for the primary reaction, except the primers were switched for the HRP3 exon 1-2 forward (3E12F) primer (5’ ATA TTA TCG CTG CCG TTT TTG CT 3’) and the HRP3 exon 1-2 reverse (3E12R) primer (5’ CTA AAC AAG TTA TTG TTA AAT TCG GAG 3’). Then 18 µl of the secondary master mix was added to each secondary reaction tube, followed by 2 µl of the 1:10 dilutions of the primary reaction products. The *pfhrp3* exon 1-2 PCR conditions for the secondary reaction were identical to those of the primary reaction except that the annealing temperature was increased to 62°C.

The *pfhrp3* exon 2 master mix was set up the same as for the *pfhrp3* exon 1-2 reactions except that for the primary reaction the primers were switched to HRP3 exon 2 forward (3E2F) primer (5’ CTC CGA ATT TAA CAA TAA CTT GTT 3’) and HRP3 exon 2 reverse primary (3E2R1) primer (5’ AGT GAT GCG TAG TGG CAT TA 3’); for the secondary *pfhrp3* exon 2 reaction, primers 3E2F1 (5’ GAA AGT CAA GCA CAT GCA GG 3’) and 3E2R (5’ GCA TTA TGG TGT GCT CCA TC 3’) were used. The primary *pfhrp3* exon 2 was carried out the same as for the *pfhrp3* exon 1-2 reaction except that the controls were diluted 1:10 prior to the primary reaction. The following PCR conditions were used for the *pfhrp3* exon 2 primary reaction: 95°C for 5 minutes, followed by 30 cycles of 95°C for 30 seconds, an annealing step of 55°C for 30 seconds, 68°C for 1 minute, followed 5 minutes at 68°C before the temperature was held at 4°C indefinitely until the samples could be retrieved. The primary PCR products were then diluted 1:200 for the unknown template DNA samples and 1:20,000 for the control DNA samples. The secondary reaction was prepared using 18 µl of the master mix per tube and 2 µl of the appropriate diluted primary PCR product. The secondary reaction conditions for *pfhrp3* exon 2 were identical to the primary reaction conditions except that the annealing temperature was increased to 57°C, and 20 cycles were used instead of 30.

For all *pfmsp1*, *pfmsp2*, *pfhrp2*, and *pfhrp3* PCRs, *P. falciparum* DNA controls 3D7, 7G8, Hb3, Dd2, and a water non-template control were used in place of the unknown genomic template DNA. All PCRs were carried out using BioRad T100 thermal cyclers (Biorad, Hercules, CA). The presence or absence of the sequence of interest in the final PCR product was confirmed by gel electrophoresis using 1.5% agarose gels stained with Gel Red Nucleic Acid gel stain (Biotium, Fremont, CA), run for 30 minutes at 170V. 1 Kb DNA ladders were included at the start and end of the gel to allow for size comparison with the sample and control bands. Gel images were captured using the Gel Doc XR+ imager with Image Lab software (BioRad, Hercules, CA). All PCR products that could not be analyzed by gel electrophoresis the same day were stored at 4°C for use the following day or at -20°C for storage longer than 24 hours.

**References**

1. Rogier E, Plucinski M, Lucchi N, Mace K, Chang M, Lemoine JF, et al. Bead-based immunoassay allows sub-picogram detection of histidine-rich protein 2 from Plasmodium falciparum and estimates reliability of malaria rapid diagnostic tests. PLoS One. 2017;12(2):e0172139.

2. WHO. Global technical strategy for malaria 2016-2030. Geneva: World Health Organization; 2015 2015.

3. Lucchi NW, Narayanan J, Karell MA, Xayavong M, Kariuki S, DaSilva AJ, et al. Molecular Diagnosis of Malaria by Photo-Induced Electron Transfer Fluorogenic Primers: PET-PCR. PLOS ONE. 2013;8(2):e56677.

4. Abdallah JF, Okoth SA, Fontecha GA, Torres RE, Banegas EI, Matute ML, et al. Prevalence of pfhrp2 and pfhrp3 gene deletions in Puerto Lempira, Honduras. Malar J. 2015;14:19.

5. Jones S, Subramaniam G, Plucinski MM, Patel D, Padilla J, Aidoo M, et al. One-step PCR: A novel protocol for determination of pfhrp2 deletion status in Plasmodium falciparum. PLoS One. 2020;15(7):e0236369.

6. Herman C, Huber CS, Jones S, Steinhardt L, Plucinski MM, Lemoine JF, et al. Multiplex malaria antigen detection by bead-based assay and molecular confirmation by PCR shows no evidence of Pfhrp2 and Pfhrp3 deletion in Haiti. Malar J. 2019;18(1):380-.
